# Pediatric oncology caregiving as narrative repair: Restor(y)ing disrupted family biographies and damaged moral identities

**DOI:** 10.1177/13634593241270955

**Published:** 2024-08-05

**Authors:** Monica L Molinaro, Jessica Polzer, Debbie Laliberte Rudman, Marie Savundranayagam

**Affiliations:** McGill University, Canada; University of Western Ontario, Canada; University of Western Ontario, Canada; University of Western Ontario, Canada

**Keywords:** illness narrative, narrative repair, pediatric oncology, proximity, storytelling

## Abstract

Drawing on Arthur Frank’s conceptualization of narrative repair, we consider how pediatric oncology nurses restore and re-story the narratives of patients and families whose biographies have been thrown off course by the diagnosis and death of a child from cancer, as well as their own narratives as caregivers. Frank argued that when one’s life story is shipwrecked by chronic or life-threatening illness, storytelling is way to reorient one’s biography to a new ending, repairing the narrative wreckage created by the illness experience. In this critical narrative study with nine pediatric oncology nurses in Ontario, Canada, we highlight how, through physical, narrative, and moral proximity, nurses become entwined in their patients’ and families’ illness narratives, and how developing this narrative knowledge provides nurses with opportunities to steer families onto new terrain. As well, we examine how nurses re-story and repair their own identities as “good” caregivers in situations when they are prevented from acting on behalf of their pediatric cancer patients. These findings contribute to literature on illness narratives by considering narrative repair as a relational process enacted as part of pediatric oncology caregiving.

## Introduction

Founded on the assumption that storytelling is central to how humans make meaning and construct and negotiate their realities and identities (Mishler, 1986, 1995; Smith and Sparkes, 2008), Frank (2013) suggests that storytelling provides a way for individuals to re-story, and thus restore, a sense of self in the face of chronic and potentially life-threatening illness. In *The Wounded Storyteller*, Frank (2013) describes the “narrative wreckage” (p. 53) that follows an individual’s diagnosis with a life-threatening illness, which irreversibly alters their life course and thereby their identity. Through this conceptualization, Frank invites us to consider how the experience of chronic and life-threatening illness inflicts damage upon and threatens the coherence and stability of one’s life story, thus throwing into question one’s preconceptions about how the future will unfold.

Just as self-narratives become “shipwrecked by the storm of disease” (Frank, 2013, p. 54), storytelling can be used to repair and reorient one’s sense of self within this altered landscape. Expanding upon the shipwreck metaphor, Frank (2013) argues that “Stories have to *repair* the damage that illness has done to the ill person’s sense of where she is in life, and where she may be going. Stories are a way of redrawing maps and finding new destinations” (p. 53, original emphasis). Rather than presume a return to a former healthy self through an overwriting or erasure of the illness experience, Frank (2013) suggests that storytelling assists individuals in constructing and navigating their new biographies. In this way, telling one’s illness story assists in forging new directions for one’s life story and creates possibilities for how the revised story might unfold.

Since Frank’s (2013) original conceptualization of narrative repair in relation to cancer narratives, narrative repair has been used to examine experiences of “strangeness” during adjuvant chemotherapy treatment for cancer (Greenhalgh, 2017), partner abuse (Barnes, 2013), breathlessness (Malpass and Penny, 2019), and injury experienced in sport (Smith and Sparkes, 2009). These studies tend to focus on and explore the narrative repair done by an individual on their self-narrative and identity after an illness, injury, or life-altering experience. However, the ways in which narrative repair might apply to the experiences of caregivers has not been previously considered.

In this paper, we move beyond individual experiences of identity disruption to consider how caregiving involves restor(y)ing family narratives and caregiver identities within the context of pediatric oncology nursing. When a child is diagnosed with an illness like cancer, it is not just an individual’s sense of self that has been shipwrecked; the narrative of the entire family has been thrown off course, and subsequently it is the entire family whose narrative must be reoriented. We argue that pediatric oncology nurses mobilize their knowledge of the patients’ and families’ narratives to steer their family illness stories onto new terrain.

In the field of pediatric oncology, nurses are understood as occupying a unique spatiotemporal position regarding a patient’s care. Nurses are often in the closest proximity to patients and their families through their care provision in both the hospital and home (Canadian Cancer Society, 2021; Pediatric Oncology Group of Ontario, 2018). This close proximity to patients and families is also established via the variety of caregiving responsibilities that nurses perform, including technical, biomedical care (e.g. treatments and procedures), as well as relational, emotional care, through which close relationships with patients and their families are developed.

In her analysis of the connections between the art and science of pediatric oncology nursing, Cantrell (2007) asserts that, in order for the tasks involved in the science of nursing practice to “be truly effective, *they must be embedded within the art* of nursing practice” (p. 132, emphasis added). In this sense, administering medication and treatments, such as chemotherapy, involves nursing presence (or physical proximity), knowledge of the patient (or narrative proximity), creativity, and resourcefulness, that are often taken for granted:

as the pediatric oncology nurse prepares the drugs to administer, she or he also considers several other critical aspects in the plan of care. The nurse considers the manner by which she or he will enter the adolescent’s room with the chemotherapy, the timing of when to enter the room, the meaning of this experience for the adolescent and his or her family, the nature of how to approach the patient, and how much interaction to have with the adolescent (how much or how little to say). (p.135)

By pointing to the embeddedness of the art and science of pediatric oncology nursing, Cantrell (2007) highlights how the interactions necessary to facilitate treatment will vary by patient, and how these interactions are dependent on how well the nurse has come to know the patient and their family. This is resonant with Malone’s (2003) insights about the three nested elements of proximity that nurses have to patients: physical proximity (being physically close to the patient and family), narrative proximity (a process through which nurses come to know the patient and their family through storytelling), and moral proximity (through which nurses recognize the vulnerabilities of the patient and are enabled to advocate on their behalf).

In this paper, we draw on Malone’s (2003) insights about the centrality of nurses’ physical, narrative, and moral proximity to patients and extend Frank’s (2013) theorization of narrative repair to consider the ways in which pediatric oncology nurses engage in narrative repair as part of their caregiving. Drawing on interviews with nine pediatric oncology nurses in Ontario, Canada, we explore how the nurses’ physical and narrative proximity to pediatric patients and families enables pediatric oncology nurses to uphold their moral identities and enact two forms of narrative repair. Specifically, we describe: (1) how the nurses’ engagement in moral proximity facilitates narrative repair for the family; and (2) how the nurses narratively restore and restory their fractured moral identities and unresolved caregiving stories. We go on to suggest that this narrative repair assisted the pediatric oncology nurses in restoring their own moral identities, which were compromised by institutional conditions that restrained their ability to enact their moral responsibilities to patients and families, and resulted in moral distress (Molinaro et al., 2023). Furthermore, we contend that this narrative repair helped mend and reorient the biographies of the families they cared for after the loss of a child with cancer. We conclude by exploring the implications of Frank’s (2013) conceptualization of narrative repair for caregiving, and suggest that it is a fruitful way of thinking about the role of restorying in providing care to those with life-threatening illness and in managing compromised caregiver identities. Before presenting these aspects and discussion of narrative repair, we provide an overview of the critical narrative methodology that guided the study from which this paper is derived.

## Methodology

The doctoral thesis upon which this paper is based sought to examine pediatric oncology nurses’ caregiving narratives (Molinaro, 2021). This study used a critical narrative methodology, which locates individual narratives and the meanings ascribed to them within larger power structures and focuses on situating and analyzing stories in relation to their cultural, social, and political contexts (Kincheloe and McLaren, 2005; Laliberte Rudman and Aldrich, 2017).

### Theoretical lenses

In this paper, we put Frank’s (2013) narrative repair in conversation with Malone’s (2003) conceptualization of proximity to draw attention to the ways in which physical proximity fosters narrative proximity between nurses, patients, and patients’ families, which then allows for nurses to engage in narrative repair as part of their caregiving and enactment of moral proximity. As well, we draw on Peter and Liaschenko’s (2013) theorization of moral distress, which encompasses moral identities (the nurses’ idealized identities), moral relationships (relationships with patients, families, and colleagues), and moral responsibilities (responsibilities to patients to act on their behalf). Elsewhere, we have illustrated how nurses use counter-stories to re-locate the sources of their moral distress within institutional constraints and dis-locate dominant narratives of technological cure by ascribing value and meaning to the relational care through which they sustain moral responsibilities with patients and their families (Molinaro et al., 2023). In this paper, we focus on how nurses attempt to repair their damaged moral identities by narratively restor(y)ing their identities as “good” caregivers who strive to uphold their moral responsibilities to patients and families.

### Data collection and analysis

All methods were approved by a university research ethics board prior to the beginning of recruitment and data collection. In total, nine nurses were recruited for this study from an email sent by two leading members of a provincial nursing group. These nurses worked in a range of locations throughout Ontario, Canada, and had 3–33 years of experience in pediatric oncology. Four of the nurses worked in inpatient (or bedside nursing) environments, three of the nurses worked in outpatient/transplant environments, and two were community travel nurses.

Following Bertaux and Kohli (1984), each nurse participated in two narrative interviews with the first author via Zoom (*N* = 15) or by telephone (*N* = 3) over a 10-month period (February to November 2020). The first interview was approached as an opportunity for the participant to engage in storytelling prompted by an open-ended question from the interviewer (i.e. “Can you please tell me, in as much detail as you can, your story of what it is like to be a pediatric oncology nurse?”), who then probed in response to this story for the remainder of the interview. The second interview was “a period of questioning” (Bertaux and Kohli, 1984, p. 224) during which participants were probed for more narrative detail about their caregiving experiences. Study participants received a copy of their interview transcript prior to the second interview, which was initiated by asking the participant if they would like to reflect on their first interview. The remainder of questions in the second interview were derived from analysis of the first interviews, and included questions specific to each participant’s narrative as well as questions to elaborate on themes that were identified across the interviews. Interviews ranged in length from 50 minutes to 3 hours and 15 minutes and were transcribed verbatim, generating roughly 400 pages of interview data. Analysis of the narratives was informed by Lieblich et al. (1998) and Laliberte Rudman and Aldrich (2017), who emphasize multiple close readings of the data, developing interpretive narrative themes, and noting contradictions, tensions, and ambivalences within the data and themes. Provisional findings were prepared by the first author for the co-authors (the advisory committee) who provided feedback throughout the course of data collection and analysis. Through this iterative process, it became clear that the interviews acted as a narrative space for the nurses to tell their caregiving stories and articulate facets of their work that may have been difficult for them to discuss elsewhere or with other audiences. As the interviewing and analysis unfolded, the authors came to understand how this narrative space facilitated the nurses’ telling of stories about the narrative repair they enacted with families, and provided an opportunity for nurses to engage in reparative story-telling to help mend their damaged moral identities as nurses.

## Results

Our analysis suggests that pediatric oncology nurses engage in narrative repair in two distinct ways. In the first section, we present stories to illustrate how the nurses engage in narrative repair as part of their care for the patient and patient’s family in situations where the patient is dying. In these instances, pediatric oncology nurses aim to steer the family onto new terrain by engaging in legacy building and facilitating meaningful death. Second, we illustrate how the nurses engage in narrative repair to mend their damaged moral identities when workload constraints and institutional demands prevented them from upholding their moral responsibilities to patients, particularly in instances where the death of their patient caused them moral distress.

### Narrative repair and caregiving: Facilitating meaningful death through narrative and moral proximity

The nurses’ narratives acknowledged that their caregiving inevitably involved forming strong relationships or “partnerships” with families, resulting from their physical proximity to patients. Through these relationships, they gained narrative proximity to patients and families who shared their stories with the nurses over time: patients’ stories of what they hope to be when they grow up; parents’ stories of what their child was like before they had cancer and of their hopes for their child; stories of grief and sadness; and stories unrelated to cancer, including stories about embarrassing moments or familial memories that made everyone laugh.

The narrative proximity that the nurses described having to patients and families was viewed by some of the study participants as a unique feature of pediatric oncology. Some nurses contrasted this narrative proximity to nursing in other pediatric environments, such as intensive care units, where the nurses “don’t know the patients at baseline” and “don’t know people’s stories” (P4, Inpatient Nurse). The importance of this proximity was revealed further when the nurses spoke about the narrative reciprocity facilitated by the long-term nature of pediatric cancer caregiving, through which nurses became thoroughly entwined in the family’s story: “Their victories, their celebrations are your victories and celebrations. Their bad times are your bad times” (P2, Inpatient Nurse). This enmeshment of the nurses in the families’ stories was highlighted by many of the nurses who storied themselves during their interviews as key characters, and at times extended family members, in the family’s pediatric cancer story:

You’re there for when the kids take their first steps, there’s this one kid [. . .] we, met her when she was eight months old and she was just learning to like, sit up, and then we saw her walking and her hair’s now back and she’s running and like blowing us kisses like, you see these kids grow up right in front of your eyes and, you feel like you were there. Like, you helped raised them you were a very big part in that, kind of like their big sister. (P3, Outpatient Nurse)

Many of the nurses’ narratives included similar stories about feeling like they were members of the family due to their physical and narrative proximity, which enabled them to witness the patient grow and change during the time they spent caring for them.

This narrative proximity and enmeshment enabled the nurses to mobilize narrative knowledge about patients and families as part of their caregiving. This was most pronounced in nurses’ stories of cases when a child was dying and where their physical and narrative proximity informed how they could provide a “good” death for the child and family. The nurses’ caregiving narratives revealed how they actively engaged in narrative repair work, which involved caregiving tasks that focused on creating meaningful memories and tangible keepsakes for the family to bring home. The nurses’ stories suggested that they facilitated these memorial activities as part of their caregiving in an effort to repair the family’s narrative, which had been disrupted by the child’s cancer and eventual demise. As an attempt to re-story that which is culturally incomprehensible (the death of a child from cancer), this narrative repair work aids in reorienting the family’s narrative in a new direction, one in which “the tidy ends are no longer appropriate to the story. A different kind of end – a different purpose – has to be discovered” (Frank, 2013: 55).

This narrative repair work was revealed in the nurses’ stories about how they were involved in what they referred to as “legacy building” activities. These activities involved creating keepsakes for the family, which provide physical testimony of the child’s life after the child died. These included “hand molds and handprints [. . .] Memories to take home” (P8, Inpatient Nurse). These memorial objects facilitate the enduring relationships between the family and child, and the nurses’ stories suggested how their work facilitates these relationships. P5 recounted how the creation of hand molds with her former patient enabled an ongoing playful relationship between her former patient and his family after he died:

I jokingly said to him, ‘when your hand’s in the mold, you’re doing whatever you want you could be making a fist or giving us the middle finger, we don’t know.’ [. . .] And, after he had died [. . .] when we were doing the molds his middle finger fell off, which like [laughs] was really funny and when we [laughs] when the parents came to pick it up, like, everyone was laughing hysterically they were like, ‘oh my gosh that’s so typical of him, like that is for sure a joke that he played from up above because, he would’ve loved for his middle finger to fall off’ [. . .] and I think the last time anyone had any communication with the family his sister was saying that any time she has to go and do something that’s like kind of hard or she needs to be really brave she takes her brother’s middle finger and puts it in her pocket [laughs] so it’s like [laughs] it’s just something that’s really funny but really unique to that family. (P5, Outpatient/Transplant Nurse)

Similar to P5’s story about the hand mold, other nurses suggested that involving the patients in the process of legacy building was an important feature of facilitating a meaningful death. This involvement included getting them to “help plan what they want at their funerals or, talk about really amazing things what heaven or whatever the afterlife looks like to them” (P1, Outpatient Nurse Manager). By having the patients speak to what they would prefer and imagine their deaths look like, the nurses enacted narrative repair by mobilizing their narrative knowledge to make these imaginings a reality and enabling a “good death”—one that the patient had a hand in constructing. This was the case for P7, whose teenaged patient wanted to create “memory boards or vision boards” and “videos and dances and pictures” to record memories of their family and life. These legacy-building items were then given to the family so that they could remember their child experiencing joy before their child died (P7, Inpatient Nurse, Interview 2). Similarly, P3’s story highlighted how her understanding of her patient’s wishes and the family’s narrative made their child’s death meaningful, particularly by making memories for the family before their child died. For her, this was an example of a “perfect” death, one that both highlights the lengths that she went to in order to support the patient and family, and enabled her to leave the family with the best narrative ending possible:

they [parents] wanted to take her to the aquarium and it wasn’t gonna happen, and there’s this wonderful window [on the unit]. And they decorated it like an aquarium they drew on jellyfish and stuff [. . .] then the next day I had her again. [. . .] I was like ‘do you guys want to do something today?’ Like, ‘let’s do something. What do you want to do.’ And they were like ‘is there like a nice room that we can sit in’ I was like ‘yes I’ve got a great idea.’ So there’s a starlight lounge on the [lower] floor. And it’s, it was newly renovated at the time [. . .] big bright windows, there’s, games in there and PlayStations and just, toys and toy kitchens whatever huge space. We went up there, and her [laughs] ENTIRE family came too. You’re not supposed to have food and drinks in there but, I turned a blind eye quite frankly I was like, no problem. [. . .] Um, anyway we had the music therapist up there, [kids cancer camp] came, all the doctors I called her doctors and I said ‘come play up here we’re just having a celebration of life’ she was on like, Midaz [a sedative medication to help with anxiety], and, I just remember, she was asleep most of the day but whenever she would wake up, the mum would look at me and, be like [whispers] ‘Is she okay? Is she okay?’ and I was like ‘Yes. Just enjoy.’ I just tried to get them to focus on her and the fun and singing the songs and, I had pressed her Midaz bolus [given her more medication] like every now and again but, most of the time was just them playing. Anyway [the] space closes at 4PM normally so I was, I’m not kidding there was like 30 people in this room at this point. I called security and I was like ‘can we keep this space open for a little bit longer’ they’re like ‘nope, we close at 4PM’ and I was like ‘listen. This kid is gunna die today or tomorrow, this is their family’s request. Please make this happen for me.’ [. . .he] called me back, and he’s like ‘yupp they can keep it as long as they want’. They just stayed in the room singing songs, playing, whatever just having a great time. Like it was literally a celebration of her life. And then at 12:30AM they decided they were tired so mum and dad took her downstairs and got into bed and put [her] between them and she died while they were asleep. And I was like, that is, perfect. Like, that is, all you can ever hope for. (P3, Inpatient Nurse)

### Narrative repair and nurses’ moral distress: Mending damaged moral identities

In comparison to the stories above, where the nurses illustrate how their caregiving involved repairing the family’s narrative through legacy building and facilitating meaningful death, the nurses’ narratives also showed how their interviews with the first author provided space for them to restore their identities as “good” caregivers in response to situations that compromised their capacity to uphold their moral responsibilities to patients and families. The distress that arose in these situations stemmed from their inability to maintain moral proximity to the patient, mobilize their narrative knowledge of patient and family, and provide care to the patient and family in a way they felt was meaningful and consistent with their ideals of what it means to be a good nurse.

This was pronounced in instances when families were unaccepting of a child’s death and when nurses were prevented from providing care that they felt would respect the patient’s wishes, facilitate a meaningful death, and assist in repairing the family’s narrative. In these situations, the nurses’ stories suggested that they were experiencing moral distress, as they struggled to maintain moral proximity to the patient and thus advocate on the patient’s behalf. These morally conflicted situations were elaborated in the nurses’ stories about patients whose Do Not Resuscitate (DNR) orders were overturned by parents who demanded cardiopulmonary resuscitation (CPR) out of desperation to keep the child alive when their heart stopped. The nurses described feeling as though they were bringing the children back to life to be in pain, which was inconsistent with their goals for care, and, in some cases, their knowledge of their patients’ wishes.

P8’s story of a girl who “had been battling cancer for eight and a half years,” and whose father could not accept that she was dying, was particularly striking. As a nurse who had provided care to the girl for most of her life (“she was diagnosed when she was two, she was ten and a half when she died”), P8’s story of the attempt to bring the girl back to life illuminates how these lifesaving measures conflicted with her moral responsibilities to the patient and her ideas about what constitutes “good” care. P8’s telling of this story was laden with despondency about the distress and moral distress associated with how she and her colleagues were unwillingly inserted into this tragic narrative, one that left lasting marks—both on the girl’s body, and on the nurses’ perceptions of the care they provided:

Dad did not want to give up [. . . .] her whole life has been cancer. [. . .] And we knew she was gonna die. Everyone knew she was gonna die but the dad never wanted to let go. So instead of giving her the pain medication that would’ve made her comfortable and letting her go to sleep and never wake up, she died while she was on the toilet. We thrust her back in bed, we did a FULL ROUND of everything so we’re like breaking her ribs ‘cause we’re doing CPR. We’re giving her the meds that she needs to get her heart beating even though it’s not beating on its own, because dad couldn’t find closure. I didn’t agree with that but it’s not my place to say it’s not my choice. [. . .] we can make death such a beautiful thing, as twisted and weird as that sounds. We can make them comfortable where their last memory is being held by their parents and singing their favourite songs or watching their favourite show cuddling in bed with mom and dad. Having their dog come and sit on their lap. We can make that their last memories before they go, or the families’ last memories of them. Not their body bruised and broken, because of us trying [to keep them alive through performing CPR]. That’s what the family wanted so that’s what we did, but I just didn’t agree. (P8, Inpatient Nurse)

In contrast to the “perfect death” narrated by P3, this story illustrates how nurses were prevented from repairing the damage of the child’s untimely death, which compromised their moral identities as nurses. At the end of the above passage, the distress experienced by P8 is contrasted with an alternative reparative narrative in which she imagines how the nurses could have enacted their moral responsibilities by facilitating a peaceful passing for the child and family. By asserting this alternative ending to the child’s life story, P8 attempts to restore her caregiving identity by illustrating how end-of-life care can help steer families onto new ground by facilitating a meaningful death—one where the child is comfortable, free of pain, and able to spend time with their family and loved ones. P8’s later disclosure that she and her colleagues, while attending the visitation, could still see the bruises on the child’s body from their resuscitative efforts was a distressing reminder of their last morally conflicted interaction with this child.

In contrast to P8’s story of how her moral proximity was constrained by parents’ desires to keep their child alive at all costs, P3 attempts to narratively repair her damaged moral identity by elaborating on her experience of the distress she experienced when she had insufficient time to tend to the specific needs of a patient who died suddenly. In this lengthy story, P3 detailed how she was unable to establish proximity to the patient and give her the care she needed because of competing and more urgent workload demands. As this emotionally fraught passage suggests, being unable to “fix” her relationship with the patient before the patient died was the source of incredible moral distress for P3, who left her position in inpatient oncology shortly after the patient died:

I had this one patient in induction she was, obviously brand new very anxious. Um, trying to get her to take steroids was just a nightmare [. . .] she was throwing them just, not cooperative I was trying to do her blood pressure and just, taking vitals took her like a half an hour and I was just like ‘I have two other kids I don’t have time for this.’ I literally said, ‘okay, why don’t you call me when you’re ready because I don’t have time for this’ [sniffles]. And anyway I pulled her mom out of the room and was like ‘I’m really sorry I’m really frustrated can you help me find a way to connect with her easier just this is not working well and I want to have a good day with her’, and she’s like [. . .] ‘she thinks you don’t care about her she thinks that you’re rushing her she thinks you don’t care about her.’ And I was like ‘I do, I so do but I’m just, tied. I’m just tight for time and I have two other kids’ and, if I had more time then I could’ve been more patient and I could’ve done things a little bit differently and I could’ve changed the way I approached it and not been so pushy but I just, I didn’t have time. Um, and my meds were late for the other kids like I just, I knew that I had, you have a set amount of time that you can be with these kids. Anyway [pause] I didn’t have her again [. . .] but, I think it was day 17 or day, under day 20 basically I was coming out of day shift and at 6:30am she called out [pauses] for a nurse saying she couldn’t see. Um [pause] and they tried to do her blood pressure and couldn’t get a blood pressure couldn’t feel peripheral pulses, she was in DIC she um, ended up having fasciitis and, she went septic from [pause] her bowels leaking and, she died. They did CPR down in ICU for fifteen minutes and she died. And I, couldn’t fix my relationship with her. [pauses, becomes emotional] I was so upset [starts crying] for so long about that [cries, pauses]. Anyway that was the night I applied to [kids cancer camp] and I took [pause] a secondment through [hospital] so I’m still gunna be on inpatient oncology unit once every two weeks but I just needed to go. I just needed a break and I felt like [sniffles] I was at the point where I couldn’t be the nurse that I wanted to be ‘cause I was so [sniffles] frustrated with the timing and the workload and I just I couldn’t be the nurse that I wanted to be and I couldn’t fix it with [sniffles] [patient]. (P3, Outpatient Nurse)

P3’s repeated articulation of “I couldn’t be the nurse I wanted to be” at the end of this excerpt illustrates how her moral identity as a caregiver was fractured by her inability to tend to the patient who was left feeling as though P3 “did not care about her.” The patient’s unexpected death meant that P3 was neither able to repair her relationship with the patient or the damage inflicted on her own moral identity, which she resolved by leaving her nursing position in inpatient pediatric oncology.

As with P8 above, P3’s caregiving story with this patient did not end with the patient’s death; it was unexpectedly continued through an encounter she had with the patient’s mother at a children’s cancer camp where she volunteered. Several nurses in this study shared their stories about how volunteering at children’s cancer camps (often using personal vacation days) enabled them to bring a sense of resolve to their caregiving stories because they were able to see children who had recovered from cancer or who had easily manageable cancers having “normal” camp experiences. In P3’s case, this story of her “serendipitous” interaction with the child’s mother provides unique insight into the value of narrative repair for nurses whose relationships with patients are fraught and left unresolved. As this passage suggests, sharing her story with the mother was reparative in the sense that it enabled her to restore her caregiver identity by making amends for the damage that she was unable to address while her daughter was alive:

This is kind of serendipitous but when I was at [kids cancer camp], [sniffles] I decided I wanted to do a [. . .] children’s grief and bereavement course. It was a five-day, certificate course. On day three [. . .] the room was kind of set up like a U. And, I heard this mum talking, saying, ‘hi my name’s [mom] I’m [pauses, sniffles] here because I’m trying to, find ways to work with my son to help him deal with my daughter’s death better. She was eleven years old.’ [. . .] and I just leaned over and I saw her face and I was like [whispers] ‘oh my god.’ [returns to normal voice] Went to the bathroom, had an absolute meltdown [pauses] and um, she was my trigger to leave [inpatient unit] like that was for me, when I realized I needed to, take a step back [sniffles]. [. . .] the whole day I couldn’t focus on the course I was like, ‘oh my god what do I do?’ [sniffles] Anyway at the end of the day I went to her and I was like ‘[name] I don’t know if you remember me but I was [patient]’s nurse’ and she was like ‘yeah of course I remember you. [daughter/patient] didn’t like you’. That’s what she said [laughs] right away. [sniffles] I was like ‘yeah I know’ and anyway I cried to [mom] and I was like ‘I just need to tell you that I really learned a lot from your daughter and [pauses] I really, took what you said to heart and I really, am trying to be more patient with anxious children because you don’t know what will happen you don’t know if you can fix it and I never want a kid to feel that I never, I didn’t care about them’. Um, so, anyway she was like ‘I’m so happy [patient] could give that to you like that’s a gift that she was able to give to you’ and, she said ‘this is meant to be’ that we like, met up in this way so that you know, we could talk, and, kind of rehash and she was asking me about, stuff, about [patient] and ‘what did this mean when they said this and this and this’ but it was so I think therapeutic for both of us in that, I really think that that was, meant to happen as a, full circle, for me. (P3, Outpatient Nurse, Interview 1)

While the research interview provided a narrative space for P3 to revisit her caregiving story and reflect on this encounter of narrative repair, her reparative narrative internalizes the blame (“trying to be more patient”) for the situation that led to her moral distress she experienced and deflects attention from the lack of time and workload demands that structured her (in)actions (Molinaro et al., 2023).

## Discussion

The critical narrative methodology used in this study allowed us to understand the meaning that nurses placed on gaining narrative proximity to their patients through their sustained caregiving relationships. Their narratives suggest the ways in which the long-term character of pediatric oncology caregiving at times involves becoming entwined in the illness narratives of patients and families, sometimes to the extent that the nurses themselves become characters in their patients’ illness stories and engage in narrative reciprocity. In this paper, we highlight the “art” of nursing practice (Cantrell, 2007) and draw attention to how the nurses’ stories enact and ascribe value to the relational aspects of nursing practice. Furthermore, our analysis provides powerful examples of how thinking narratively provides important insights into the broad scope of how nurses engage in caregiving for patients who are chronically and/or terminally ill.

Our findings extend Frank’s (2013) focus on illness narratives by illustrating how narrative repair is engrained within pediatric oncology caregiving as a way of restor(y)ing family and caregiver identities, particularly in situations of terminal illness and death. Alongside Malone’s (2003) theoretical insights about the nested character of physical, narrative, and moral proximity in nursing, Frank’s (2013) notion of narrative repair highlights the narrative aspects of relational caregiving whereby nurses actively engage patients and families in reparative narrative practices, such as creating memory boards, as patients near the end of life. As our findings illustrate, narrative repair (Frank, 2013) is a fruitful way to understand how the nurses enact relational care by mobilizing the knowledge gained through their narrative proximity to patients and families to facilitate meaningful death for patients, as well as for their parents and other family members. We also suggest that narrative repair allows for a compelling way to understand how nurses restore and re-story their moral identities in situations where their duties to their patients, and thus their professional identities as nurses, have been compromised.

In this study, narrative repair emerged both in the *content* and in the *telling* of the nurses’ stories. This narrative repair work was particularly evident in stories about providing care before, during, and after a child’s death, that is, in situations where the endpoints of the biomedical success story (remission and survival) could not be realized. Through their sustained proximity to patients, the nurses mobilized their narrative knowledge to help families navigate an unforeseen ending to their child’s life and story, steered the family onto new narrative terrain, and aimed to help families reorient their biographies in light of the narrative “wreckage” left by the child’s death from cancer. The nurses experienced moral distress when they were unable to engage in this narrative repair for the family, for example in situations where the child died suddenly or the family was not accepting of their child’s death. In such instances where nurses were prevented from facilitating meaningful death and maintaining moral proximity to patients and families, they were not able to uphold their moral responsibilities to their patients and experienced fractures to their moral identities, which resulted in moral distress (Peter and Liaschenko, 2013). Through the telling of their stories in the interviews, these nurses engage in narrative repair to restore their identities as a “good nurse,” as the nurses they “want to be” (Molinaro et al., 2023).

In addition to the ways in which narrative repair functioned as a form of relational caregiving, the nurses’ tellings of moral distress and dis-ease acted as a form of counter-story (Molinaro et al., 2023; Nelson, 2001; Peter and Liaschenko, 2013) that drew attention to the ways in which they sought to mend damage to their moral identities through narrative means. As nurses’ moral identities are created narratively, they can also be repaired narratively (Lindeman, 2006; Peter and Liaschenko, 2013). In combination with Peter and Liaschenko’s (2013) theoretical insights about moral identities and counter-stories, Frank’s (2013) notion of narrative repair has interesting implications for the ways in which nurses may enact and repair their threatened moral identities (Nelson, 2001; Peter and Liaschenko, 2013). As we have explored elsewhere (Molinaro et al., 2023), the nurses’ counter-stories act as a form of resistance that draws attention to the holistic, relational care that is necessary to sustain their moral identities, maintain moral proximity to their patients, and construct themselves as “good nurses.” Our findings illustrate that the nurses in our study restor(i)ed their moral identities by locating their moral distress in their dis-ease of working in situations where their capacities to uphold their moral responsibilities and relationships to and with patients have been constrained (Molinaro et al., 2023). In this regard, the nurses’ stories drew attention to the moral dilemmas they experienced in instances when parental desire (e.g. to continue lifesaving measures) conflicted with the wishes of their child or with what the nurses believed was in the best interest of the child. Such conflicts echoed and exacerbated the institutional constraints and caregiving demands that we have discussed elsewhere (Molinaro et al., 2023) and that restricted the nurses’ capacities to engage in narrative and moral proximity to patients and patients’ families (Molinaro et al., 2023). The nurses’ stories also revealed how they addressed unresolved caregiving narratives when their relationships with patients and families came to an end, sometimes suddenly (e.g. because of a child’s remission or death).

We suggest that, through such tellings, the research interviews provided a narrative space in which the nurses were able to restore and restory their caregiving narratives in an attempt to heal fractures in their moral identities. The positioning of the first author as a non-nurse “outsider” seemingly enabled her to provoke stories of moral distress by the nurses, or tellings that the nurses may not have shared otherwise. These findings suggest a need for institutionally recognized spaces where nurses and other health care providers can share their stories in order to expand the narrative resources available to them, as well as to patients, families, and other formal and informal caregivers, as they struggle to provide quality care in resource-constrained settings and for families whose stories must be reimagined after the death of a child. The benefits of spaces created for pediatric oncology nurses to share stories of grief have been documented (Macpherson, 2008) and narratively informed writing practices, such as reflective journaling, have also been found to be beneficial for nursing students working in pediatric oncology (Mirlashari et al., 2017). Such spaces for the possibility of narrative repair through the sharing of stories can be viewed as sites of narrative re-construction and, potentially, as sites where nurses’ narratives mediate collective experiences and collective action to advocate for institutional change and prioritize the wellbeing of caregivers who can uphold their moral responsibilities to patients and families.

## Conclusion

Due to their sustained physical, narrative, and moral proximity to their patients, pediatric oncology nurses become active characters in a family’s cancer story. The stories in this study illustrate that narrative repair is a relational process and generative way of understanding how pediatric oncology nurses attempt to repair a family’s fractured and disoriented narrative by actively mobilizing their narrative knowledge of their patients and families to facilitate meaningful death. Narrative repair further allows an understanding of how caregivers restory and restore their own identities in situations where their moral duties to patients, and their moral identities as nurses, have been jeopardized. Through the use of a safe narrative space to tell stories of narrative repair, such as through a research interview or other narrative spaces, narrative repair through sharing stories can be viewed as a collective re- and co-construction, or a collective act of repair and action.
